# IL-33/ST2L signaling alleviates diabetic nephropathy by regulating endoplasmic reticulum stress and apoptosis

**DOI:** 10.1186/s12882-023-03415-8

**Published:** 2023-12-05

**Authors:** Teng Liu, Yu-qing Jin, Qi Wang, Cong-hui Jia, Wei-yan Ren, Jia-yi Liu, Lei Yang, Hong-min Luo

**Affiliations:** 1 Department of Epidemiology and Statistics, School of Public Health, Hebei Key Laboratory of Environment and Human Health, Hebei Medical University, Shijiazhuang, China; 2 Institute of Pediatric Research, Children’s Hospital of Hebei Province, Shijiazhuang, China; 3 Department of Nephrology, Third Hospital, Hebei Medical University, Shijiazhuang, China

**Keywords:** Diabetic nephropathy, IL-33, ST2, Endoplasmic reticulum stress, Apoptosis

## Abstract

**Objective:**

Diabetic nephropathy (DN) is a serious chronic complication of diabetes mellitus (DM). Endoplasmic reticulum (ER) stress is an important factor in the regulation of pathological processes in DN, and excessive ER stress can lead to apoptosis. Although the IL-33/ST2 axis is known to be involved in diabetic kidney disease or related nephropathy, its role and molecular mechanisms remain poorly understood in terms of DN. The purpose of this study was to investigate the effects of IL-33/ST2 signaling on DN and to characterize the roles that ER stress and apoptosis play in DN.

**Methods:**

To investigate this study, mice were randomly assigned into DN (induced by 0.1% STZ) and Control groups. Biochemical indices (FBG, BUN, UPR, UCE) were measured in serum and urine samples to reflect blood glucose and kidney damage. Quantitative real-time PCR, western blot, and immunofluorescence were used to assess gene and protein expression of the IL-33/ST2 axis and ER stress relative signaling molecule. Apoptosis was analyzed by flow cytometry.

**Results:**

IL-33 levels are significantly increased in the kidneys of patients and mice with DN. Double immunofluorescence staining showed that IL-33 colocalized with CD31-positive endothelial cells. Treatment with IL-33 attenuated kidney injury in Streptozotocin (STZ)-treated mice. In vitro, we showed that IL-33 attenuated ER stress and apoptosis in glomerular endothelial cells. However, sST2 treatment significantly reversed these effects of IL-33.

**Conclusion:**

Together, these data suggest that IL-33/ST2 signaling mitigates STZ-induced renal damage, partly at least, by suppressing ER stress and apoptosis. Therefore, IL-33 may be an effective therapeutic target in DN.

**Supplementary Information:**

The online version contains supplementary material available at 10.1186/s12882-023-03415-8.

## Introduction

Diabetic nephropathy (DN), which is a severe microvascular complication of diabetes mellitus (DM), remains the principal cause of end-stage renal disease (ESRD) [1]. Glomerular injury has long been recognized as a key pathogenesis of DN, and multiple factors, such as an increased high glucose (HG) level, inflammation, oxidative stress, and apoptosis, are involved in the pathological mechanisms of DN [2]. Currently, conventional therapeutic strategies have been used for the prevention and treatment of DN, including strategies to control glucose and blood pressure and strategies to lower lipid levels, but these approaches do not always prevent the ultimate progression to DN [3, 4]. Therefore, novel therapeutic methods are crucial to prevent and inhibit the development of DN. Several studies have suggested that the development of diabetic and nondiabetic glomerular injury is associated with endothelial dysfunction [5, 6].

Interleukin-33 (IL-33) belongs to the IL-1 family and binds to the suppression of tumorigenicity 2 (ST2) receptor, inhibiting tumorigenicity [7]. ST2 has two major isoforms: a transmembrane form (ST2L) and a soluble form (sST2) [8]. The IL-33/sST2 interaction has the potential to block cellular functions when IL-33 binds to the receptor complex (comprised of ST2L and IL-1 receptor accessory protein) [9, 10]. Components of the IL-33/ST2 axis are constitutively expressed in endothelial and epithelial cells [11], which participate in the pathophysiological processes of inflammation, vascularization, and apoptosis [8, 12, 13]. The IL-33/ST2 axis has been implicated in renal diseases, including DN, in both clinical and animal studies. Some studies have shown that the expressions of IL-33 and ST2 are elevated in various renal disease models [12, 13]. It remains unclear, however, what role IL-33 plays in DN.

Researchers have been studying the underlying mechanisms of DN development and progression for the past decade. A growing body of evidence suggests that Endoplasmic reticulum (ER) stress regulates the pathological processes of DN [14]. Studies have shown that in diabetic kidney disease, hyperglycemia, proteinuria, and increased free fatty acids can trigger the unfolded protein response in cells to induce endoplasmic reticulum (ER) stress and ultimately lead to apoptosis [15]. ER homeostasis depends on ER stress, which is engaged in protein synthesis, folding, calcium storage, and signaling. Imbalanced homeostasis causes the accumulation of unfolded and misfolded proteins inside the ER and subsequently activates ER-localized transmembrane proteins, such as ATF6, IRE1, and PERK, which induce ER stress [15–17]. In addition, when excessive ER stress persists, CHOP transcriptional expression, JNK pathway and caspase-12 are activated, promoting apoptosis [18, 19].

Recently, increasing evidence has suggested that glomerular endothelial cell (GEC) damage is already present at the initiation of podocyte injury. Studies have shown that IL-33 can modulate ER stress [20, 21]. A related study reported that IL-33 improved diabetic cardiomyopathy by inhibiting ER stress-related cardiomyocyte apoptosis [21].

**Fig. 1:**
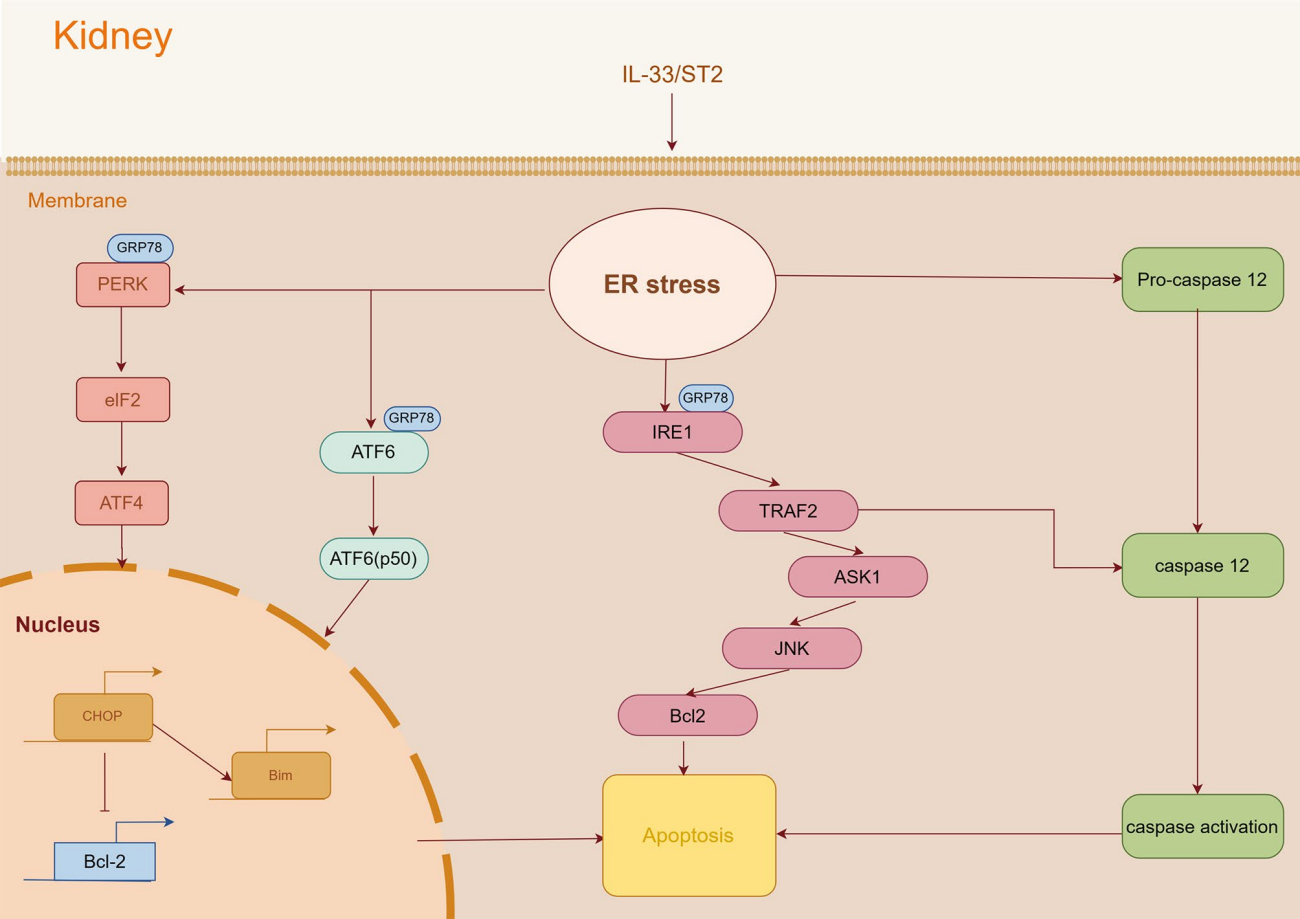
The mechanism diagram for this article

However, due to various factors such as the difficulty of simulating the chronic course of human DN in existing animal models and the long study period, there is still a lack of discussion on whether the regulatory effect of IL-33 on ER stress is related to DN process. In summary, this study applied DN animal model and cell model (high glucose) to study the effect of IL-33/sST2 pathway on DN, so as to test the hypothesis that IL-33/sST2 pathway is involved in the pathological mechanism of DN and regulates renal apoptosis by inducing ERS. The mechanism diagram for this article is shown in Fig. 1.

## Methods and materials

### Collection of gene expression dataset

We downloaded a dataset of glomerular transcriptional profiles of DN patients from the Gene Expression Omnibus Database (www.ncbi.nlm.nih.gov/geo/). The dataset (GSE96804) includes transcriptomes of glomerular tissues extracted from the kidneys of DN patients and control glomerular tissues from healthy living donors, including 42 patients with DN and 19 controls. We screened out the data related to IL-33 and sST2 gene expression.

### Animals

We obtained male C57BL/6J mice from Beijing Vital River Laboratory Animal Technology Co. Ltd. Mice aged 6 to 8 weeks and weighing 18 ~ 20 g. Animals were cared for and used in accordance with the Guidelines for Care and Use of Laboratory Animals. The Local Committee on Animal Care Use and Protection of Hebei Medical University approved the experiments.

### Experimental design and animal model

The experimental mice were maintained at a room temperature of about 22℃, humidity of about 45%, and a circadian rhythm of 12 h of light/12 h of darkness, and acclimatized feeding for 1 week [22, 23].

Forty-eight mice were randomly assigned to the DN (n = 36) and control (n = 12) groups. All mice were fasted for 12 h. The DN mouse model was established by intraperitoneal injection of streptozotocin (0.1% STZ, 100 mg/kg) on 3 consecutive days [24]. The modeling was established in 30 min with an injection of STZ (STZ was dissolved in 0.1 mol/L of citrate buffer (pH 4.5) before use) into the abdominal cavity. The control group received an equivalent volume of PBS by intraperitoneal injection. After STZ injections were administered over 72 h, fasting blood glucose (FBG) levels were measured after the mice had fasted for 12 h, and the animals were considered diabetic when the FBG level was ≥ 11.1 mmol/L. Urine samples were collected after two weeks. Urinary creatinine excretion (UCE) and random urine protein (UPR) were measured, and the urine protein creatinine ratio (UPC) was calculated. All the values were significantly increased, suggesting the successful establishment of the DN model. Thirty-six mice in which the model was successfully established were randomly divided into three groups, namely, the STZ group, the STZ + IL-33 group, and the STZ + IL-33 + sST2 group. The STZ + IL-33 group was given 14 g/kg intraperitoneally of IL-33 and STZ + IL-33 + sST2 group was given 16 g/kg intraperitoneally of sST2 every day for six consecutive days.

### Sample collection

All experimental mice were euthanized after anesthesia. The specific measures of euthanasia were as follows: all experimental mice were intraperitoneally injected with pentobarbital sodium (150 mg/kg).Urine samples and blood samples were collected and centrifuged at 3500 r for 15 min. The supernatants were stored at -80 °C. The blood was collected by extirpating the eyeball, and the samples were centrifuged at 2000 r for 10 min to obtain serum, and then stored at -80 °C. Kidneys were harvested after euthanasia, and we fixed one kidney with 4% paraformaldehyde for histological analysis and stored the other at -80 °C until use.

### Renal function analysis

Biochemical indices, including blood urea nitrogen (BUN), UPR, and UCE, were measured in the serum and urine samples using commercial kits (IDEXX Laboratories, Westbrook, ME, USA), and the results reflected the degree of kidney damage.

### Cell culture

Mouse glomerular endothelial cells (MGECs) were cultured in RPMI 1640 medium (HyClone) containing 10% FBS (HyClone), 100 U/mL penicillin, and 100 µg/mL streptomycin placed in a 5% CO_2_ incubator at 37 °C. The cells were first treated with IL-33 (10 ng/ml or 100 ng/ml, Sino Biological, 10,368-HNAE) or sST2 (Sino Biological, 10,105-H08H) for 24 h and then treated with 30 mmol/l glucose (high glucose; HG) for 24 h.

### Quantitative real-time PCR analysis

We extracted total RNA from tissues using a Promega total RNA isolation kit (Promega, Madison, WI) and reverse transcribed it with a High-Capacity cDNA Reverse Transcription Kit (Thermo Fisher Scientific, MA, USA). A Bio-Rad iQ5 real-time PCR detection system with SuperReal PreMix (SYBR Green) (TIANGEN, Beijing, China) was then used to perform quantitative real-time PCR. The ∆∆CT method was used to determine the relative changes in gene expression normalized to mouse GAPDH gene expression. The primers used are listed in Table 1.

**Table 1:** Primer list

Gene	Primer	
IL-33	Forward	GATGGGAAGAAGGTGATGGGTG
	Reverse	TTGTGAAGGACGAAGAAGGC
sST2	Forward	TTACCCAGCCAGGATGTTTC
	Reverse	CTAGGGGCTTGGCTTCTCTT
ATF6	Forward	TCGCCTTTTAGTCCGGTTCTT
	Reverse	GGCTCCATAGGTCTGACTCC
PERK	Forward	GTCGGAGACAGTGTTTGGCTTAG
	Reverse	GCGATTCGTCCATCTAAAGTGCT
IRE1	Forward	GCAGCCTGTATGTCTTGGGAA
	Reverse	TGAGTAGCGGCGATAGGTTGT
GAPDH	Forward	TGTTTCCTCGTCCCGTAGA
	Reverse	GATGGCAACAATCTCCACTTTG

### Western blotting

MGECs were homogenized and lysed with RIPA lysis buffer (Beyotime, Shanghai, China). We quantified total proteins using a BCA protein assay kit (Beyotime, Shanghai, China), separated by SDS‒PAGEand then transferred to polyvinylidene difluoride membranes. The blots were incubated with primary antibodies against the following target proteins: GRP78 (1:1000; ABclonal), Caspase-12 (1:1000; ABclonal), CHOP (1:1000; ABclonal), PERK (1:1000; Affinity), p-PERK (1:1000; Affinity), IRE1α (1:1000; Affinity), and p-IRE1α (1:1000; Affinity). The membranes were then washed with PBST (3 × 10 min) before incubation with horseradish peroxidase-conjugated secondary goat anti-mouse or goat anti-rabbit antibodies (1:5000) in 5% milk–PBST for 1 h. After another PBST (3 × 10 min) wash, the blots were incubated in the chemiluminescent substrate for 1 min and finally exposed to X-ray film (ChemiDoc™XRS+, Bio-Rad).

### Flow cytometry

An Annexin V-FITC/PI Apoptosis Detection kit (Vazyme, Nanjing, China) was used to analyze apoptosis in MGECs cultured in six-well plates. Following treatment with IL33 or sST2 for 24 h, the cells were treated with HG for 48 h. The treated cells were collected, washed with PBS (1000 r for 5 min), and resuspended in 100 µl of binding buffer. Five microliters of Annexin FITC and 5 µl of PI were added successively, and the samples were incubated in the dark at room temperature for 10 and 15 min, respectively. All the samples were analyzed using a cytometer (CytoFLEX S, Beckman Coulter).

### Immunohistochemistry

Histological examination of the kidneys was conducted after the kidneys were fixed overnight in 4% paraformaldehyde, embedded in paraffin, and cut into 5-mm sections. By incubating the sections with 3% bovine serum albumin in PBS, nonspecific binding was blocked, and endogenous peroxidase was quenched by 3% hydrogen peroxide. The sections were immuno-stained with anti-CHOP and anti-caspase 3 primary antibodies, followed by secondary antibodies (R&D, MN, USA), and the pathological changes were observed under an ordinary light microscope.

### Double immunofluorescence staining

Sections were treated with EDTA antigenic repair buffer (pH 9.0) for antigenic repair, and to quench autofluorescence, added 3% BSA, and incubated for 30 min at room temperature; then stained with an anti-rat CD31 primary antibody, an anti-rabbit IL-33 primary antibody overnight at 4 °C. Anti-rat Alexa Fluor 488 and anti-rabbit CY3 secondary antibodies were used. The cells were incubated at room temperature in the dark for 50 min, and the nuclei were stained with DAPI in the dark for 10 min at room temperature. Anti-fluorescence quenching tablets were sealed and the samples were observed and images were collected by confocal microscopy.

### Statistical analysis

All the data were analyzed using IBM SPSS software. The values are presented as the mean ± SEM. Student’s t-test was used to analyze experiments with two groups. Statistical analyses were conducted using a one-way ANOVA followed by Tukey’s multiple comparison test to determine the significant differences when more than two groups were involved. A P value less than 0.05 was considered significant.

## Results

### IL-33 and sST2 expression changed in DN

**Fig. 2:**
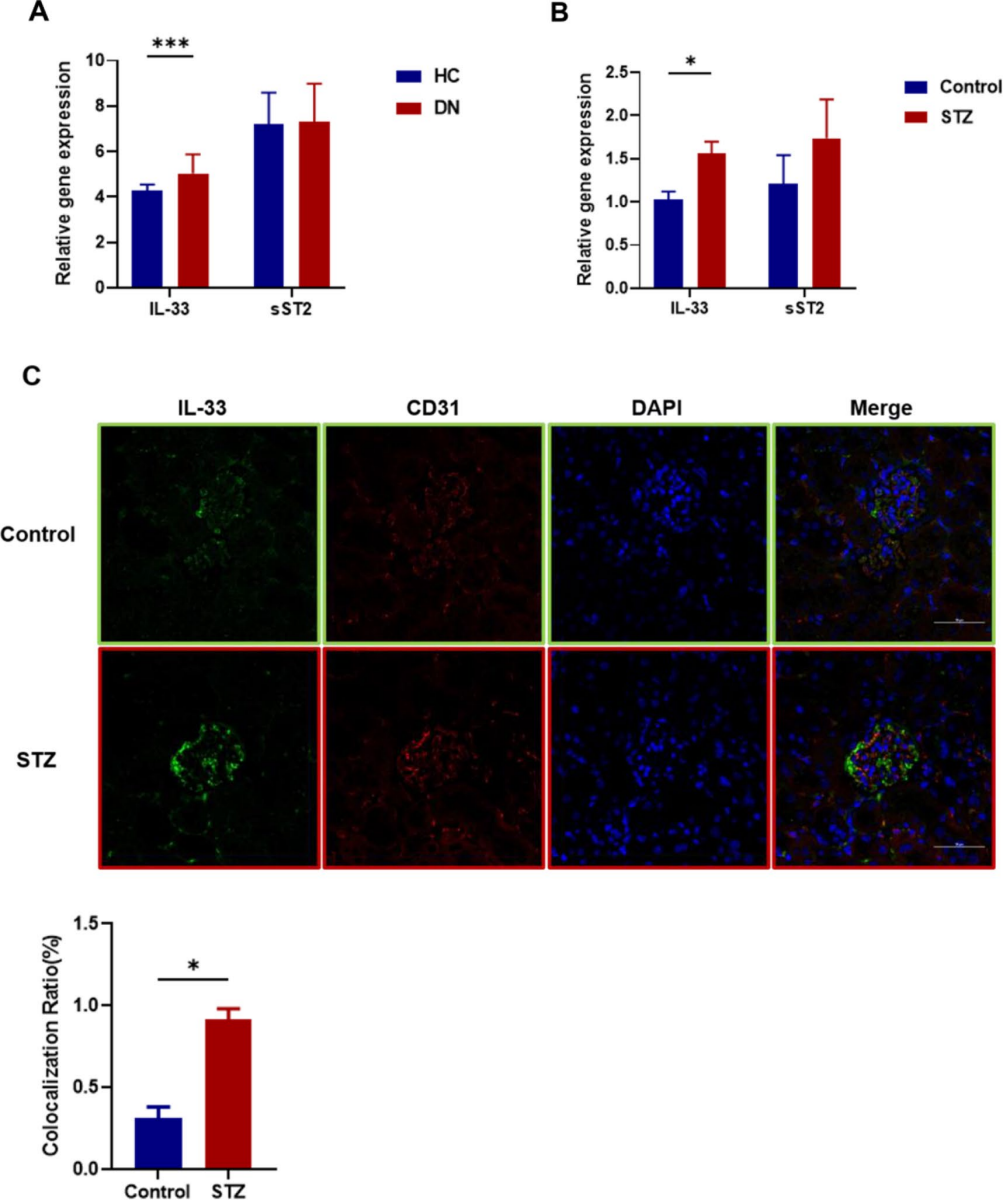
The expression of IL-33 and sST2 in DN. (**A**) Identification of IL-33 and sST2 from GEO datasets (GSE96804) in heath control (HC; n = 19) and DN patients (n = 42). (**B**) Quantitative real-time PCR analysis of IL-33 and sST2 in STZ-induced mice (n = 6 each). The expression level in the Control group was assigned a value of 1. Data are expressed as means ± SEM. **p < 0.01. (**C**) Double immunostaining with anti-CD31 and anti-IL-33 in kidney sections. Scale bars = 50 μm. Fluorescence colocalization ratio analysis showed that the expression of IL33 in glomeruli was significantly increased in STZ mice

We searched the GEO database, and a transcriptome comparison dataset (GEO: GSE96804) revealed that IL-33 expression was upregulated in DN patients (Fig. 2A). We then compared IL-33 and sST2 gene expression between control and STZ-treated mice. The data showed significantly increased IL-33 gene expression in STZ-treated mice compared to control mice. The sST2 gene exhibited a trend of elevated expression, but its expression was not significantly different between the two groups of either patients or mice (Fig. 2B).

By utilizing co-fluorescence staining, we then determined the expression and localization of IL-33 in cells (Fig. 2C). The results showed that IL-33 partly colocalized in CD31-positive endothelial cells. The ratio of fluorescence colocalization of IL-33 to CD31 for STZ group was significant higher than Control.

### IL-33 attenuated renal injury in mice with STZ-induced DN

**Fig. 3:**
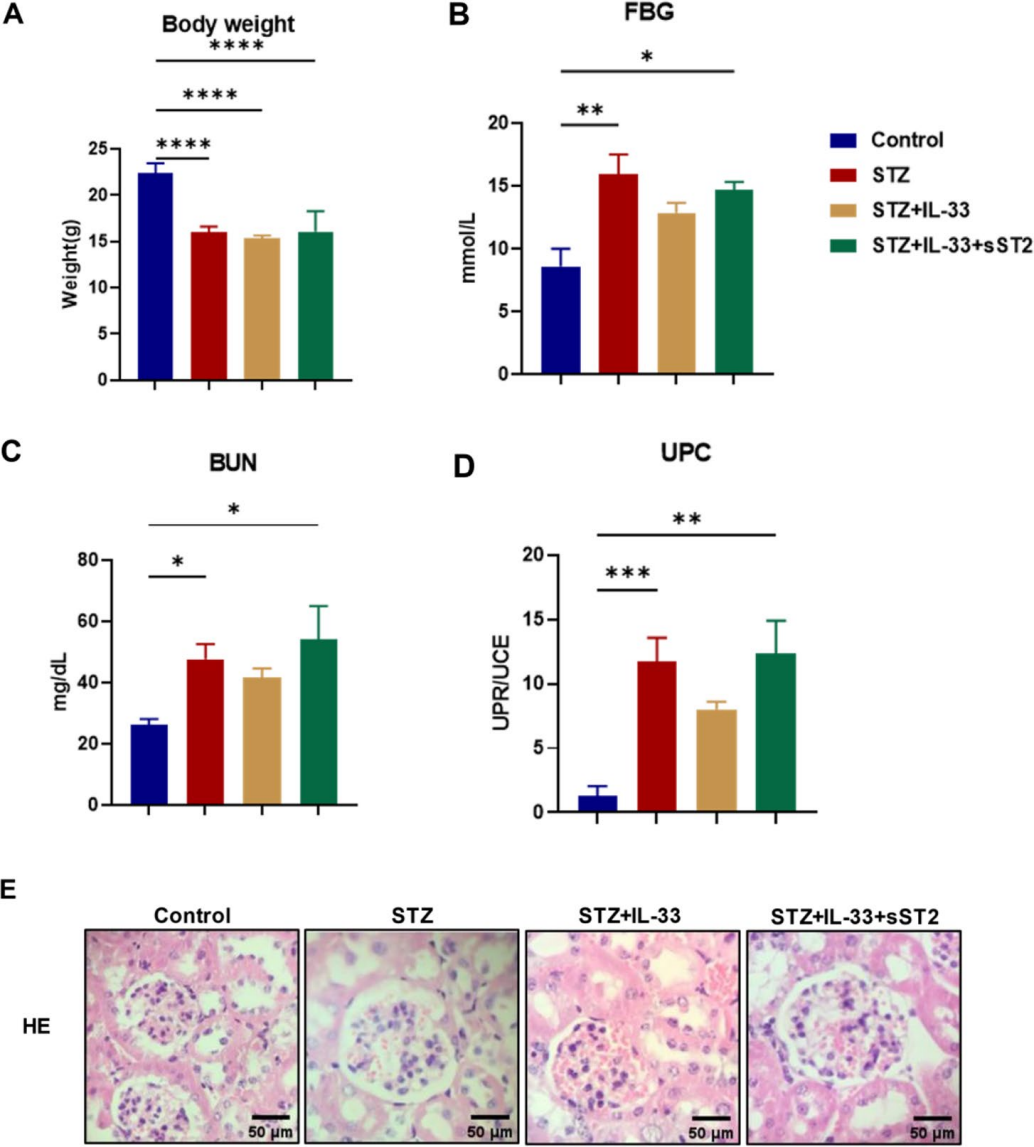
Body weight, Fast blood glucose, and biochemical indices in Control, STZ, STZ + IL-33, and STZ + sST2 mice. Body weight (**A**) and Fast blood glucose (**B**) was measured before sacrificed and expressed as the mean ± SEM. n = 6 in each group. (**C**) Serum blood urea nitrogen level. (**D**) UPC ratio: urine protein / urine creatinine. Data are expressed as means ± SEM. n = 5 in each group. *p < 0.05, **p < 0.01, ***p < 0.001, **** *p* < 0.0001. (**E**) HE staining of renal tissue. Scale bars = 50 μm

In addition, we compared the changes in body weight among the control, STZ, STZ + IL-33, and STZ + IL-33 + sST2 groups. The STZ, STZ + IL-33, and STZ + IL-33 + sST2 groups exhibited significantly lower weight gain than the control group (Fig. 3A). The statistically significant difference in FBG levels only between the control and STZ groups indicated the success of model establishment (Fig. 3B). In addition, we assessed the effect of IL-33 on renal function by measuring BUN and the UPC ratio in mice. The BUN level was significantly elevated in the experimental mice compared to the control mice, with a trend of decreasing BUN levels in the STZ + IL-33 group, while the BUN levels in the STZ + IL-33 + sST2 group increased again, showing a reversal of this trend (Fig. 3C). Significant differences in the UPC ratio were observed not only between the experimental and control groups but also among the experimental groups. In detail, the UPC ratio was markedly elevated in STZ-treated mice and inhibited by IL-33 treatment, and sST2 treatment significantly reversed the inhibitory effect of IL-33 (Fig. 3D). The application of HE staining revealed that, in comparison to the control group, the kidney glomerulus of the STZ group exhibited an augmentation in volume, vacuolar degeneration, and mesangial hyperplasia. IL-33 treatment exhibited amelioration in renal pathology, while sST2 displayed a resemblance to the STZ group (Fig. 3E).

### IL-33 suppresses the induction of ER stress and apoptosis in mice with STZ-induced DN

**Fig. 4:**
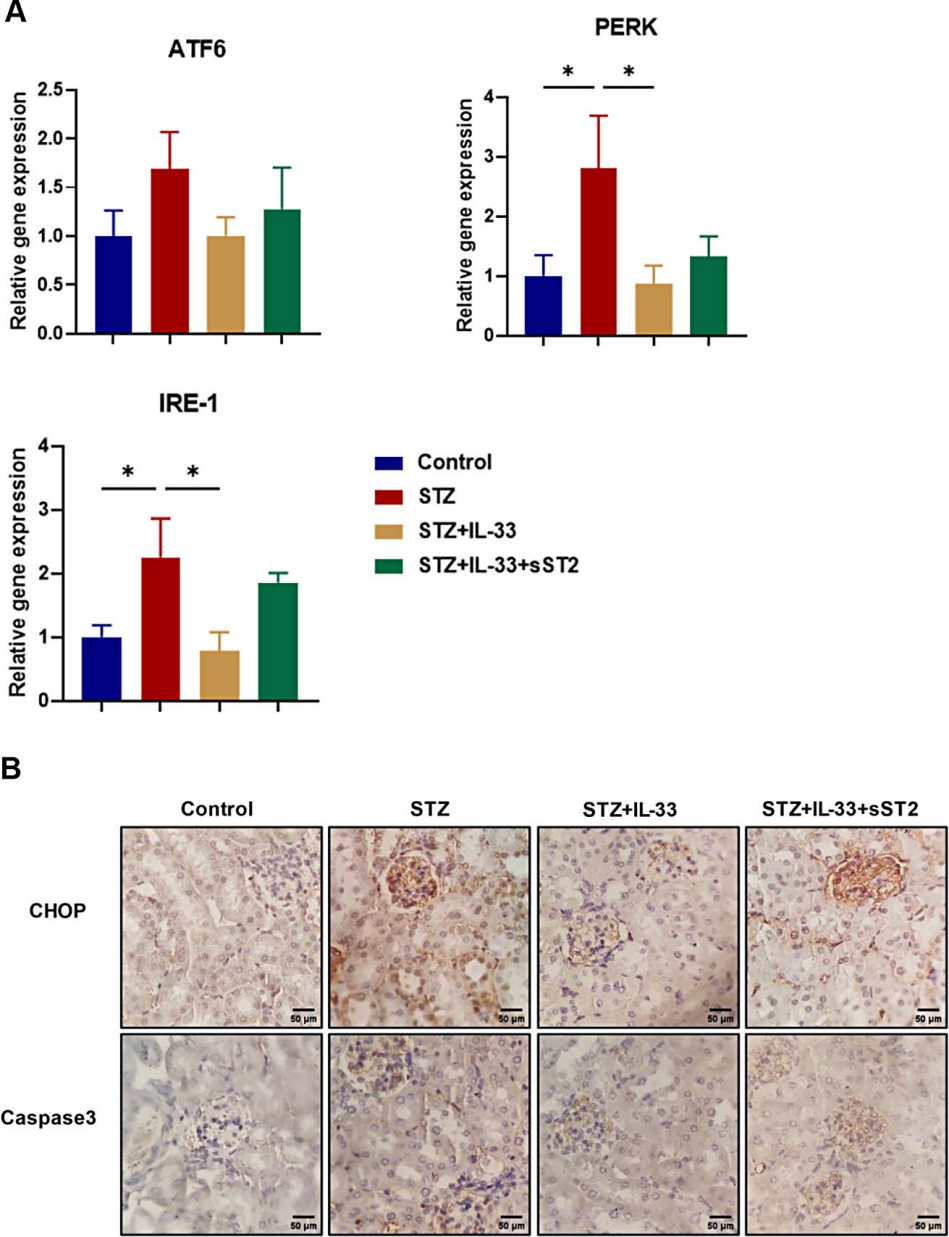
ER stress-related factors in Control, STZ, STZ + IL-33, and STZ + sST2 mice. (**A**) Quantitative real-time PCR analysis of ATF6, PERK and IRE1 in renal tissue (n = 6 each). The expression level in the Control group was assigned a value of 1. Data are expressed as means ± SEM. *p < 0.05, **p < 0.01. (**B**) Immunohistochemical staining of CHOP and caspase3 in renal tissue. Scale bars = 50 μm

We then investigated the effect of IL-33 on DN-induced ER stress and apoptosis. We first examined the gene expression of ER stress markers in the kidneys of STZ-treated mice. The gene expression of PERK and IRE1, ER stress signaling molecules, was markedly increased in STZ-treated mice, while PERK and IRE1 were significantly decreased after treatment with IL-33 (Fig. 4A). There was no further significant effect on ATF6 expression in STZ-treated mice that were also treated with IL-33. Immunohistochemistry showed that renal tissue CHOP expression was upregulated in STZ-treated mice, and this effect was attenuated by IL-33 (Fig. 4B). We then explored the effects of IL-33 on glomerular apoptosis. Apoptosis in renal tissue was assessed by immunohistochemical staining for caspase-3, and the results showed that caspase-3 was enriched in the kidneys of STZ-treated mice, while the enrichment of caspase-3 was attenuated in IL-33-treated mice (Fig. 4B). However, coadministration with sST2, a fusion protein that neutralizes IL-33 activity by acting as a decoy receptor, significantly reversed these effects of IL-33 (Fig. 4A and B).

### IL-33 attenuated high glucose-induced ER stress and apoptosis in MGECs

**Fig. 5:**
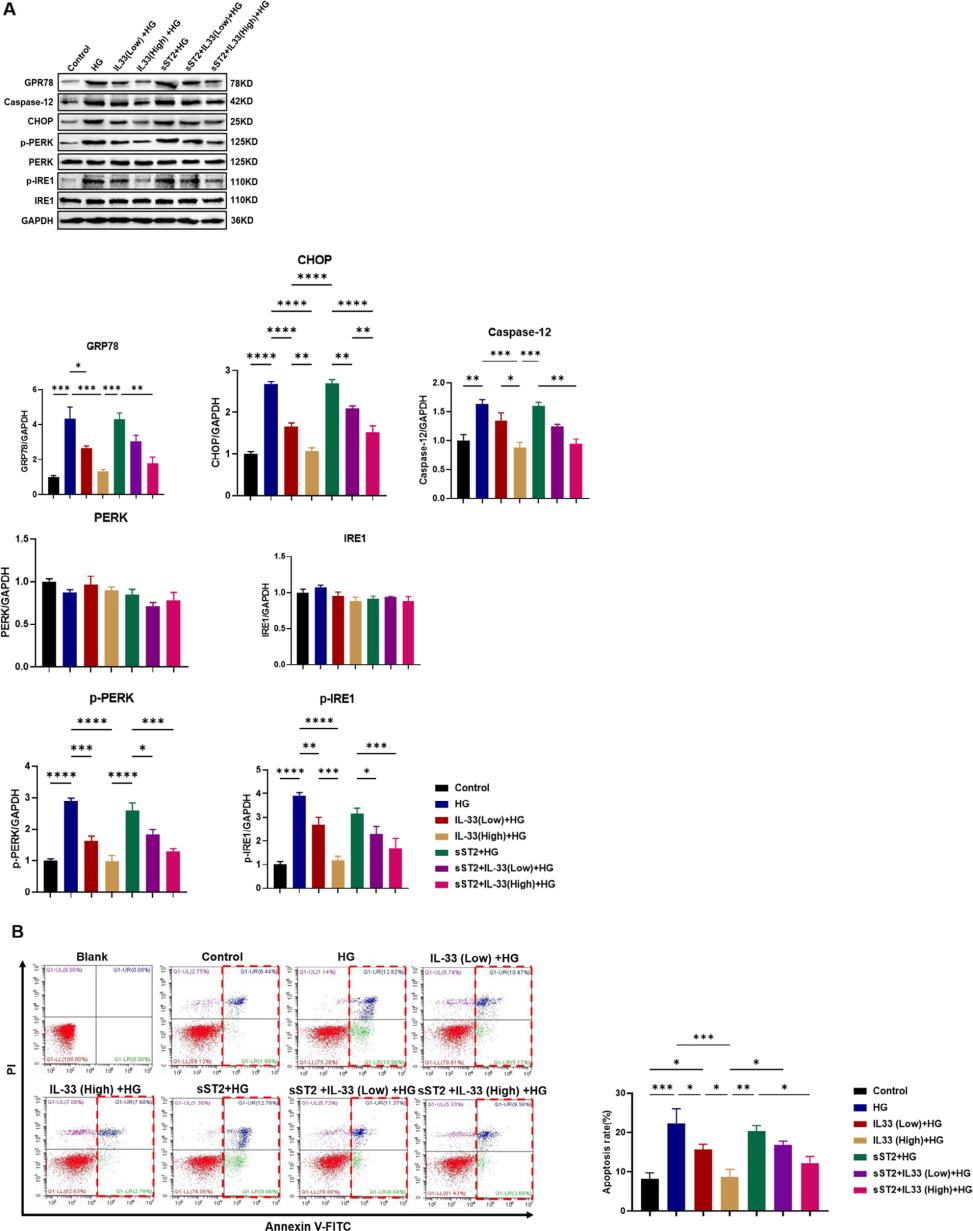
Effects of IL-33 and ST2 on ER stress and apoptosis in MGECs exposed to HG. (**A**) Western blots of MGECs showing the effect of IL-33 and ST2 exposed to HG. GAPDH was used as a loading control. Quantification relative to GADPH level. Results are representative of 1 in 3 replications. Data are means ± SEM. *p < 0.05, **p < 0.01, ***p < 0.001, **** *p* < 0.0001. (**B**) Flow cytometry analysis of apoptosis in MGECs. Representative images and apoptotic rates in cells show that a high concentration of IL-33 stimulation can reduce apoptosis. UR = late apoptotic cells; LR = early apoptotic cells; UR + LR = total apoptotic cells. The experiment was repeated at least three times. Data are means ± SEM. **p < 0.01, ***p < 0.001

MGECs were cultured with normal glucose (Control) and high glucose (HG) for 24, and the effect of high glucose on cell ER stress and apoptosis was monitored. We first examined the protein levels in MGECs by western blotting. As shown in Fig. 5, the expression levels of GPR78, caspase12, CHOP, p-PERK, and p-IRE1 were significantly increased in the HG-treated group compared with the control group. Moreover, IL-33 treatment significantly decreased the expression of these proteins in a dose-dependent manner. However, when co-incubated with sST2, the inhibitory effect of IL-33 on ER stress-related factors was reversed (Fig. 5A). The raw data can be seen in Figure S1.

To further investigate these phenomena, we explored the effects of IL-33 on glomerular endothelial cell apoptosis. Flow cytometric analysis illustrated that HG significantly increased the apoptosis rate of cells, and IL-33 treatment markedly reduced HG-induced cell apoptosis in a dose-dependent manner (Fig. 5B). Additionally, in MGECs pretreated with sST2, we found that the IL-33-mediated inhibition of HG-induced apoptosis was completely abolished. However, HG-induced apoptosis was not inhibited in HG-treated cells stimulated with sST2 alone (Fig. 5B).

## Discussion

Diabetic nephropathy is a chronic kidney disease characterized by glomerular damage that is caused by diabetes-related complications. At present, exploring the mechanism underlying the promotion of glomerular injury in DN and seeking new therapeutic strategies have become a focus of endocrinological investigations. The IL-33/ST2 pathway has been described as a potential therapeutic target and biomarker in several diseases [25]. Multiple studies have suggested that diabetic inflammation and microvascular complications are responsible for elevated IL-33 levels, and the role of IL-33 in DN remains controversial [26, 27]. On the one hand, it has been reported that IL-33 can attenuate renal inflammation and fibrosis, reduce oxidative stress, and improve renal function in animal models of DN [28]. On the other hand, some studies have reported increased expression of IL-33 in the patients with DN, suggesting a potential role in disease progression [29]. Therefore, the current study aimed to investigate the effect of IL-33 on DN and the underlying mechanisms.

In our study, we initially determined the expression of IL-33 and sST2 in DN patients from the GSE96804 dataset. Then, we explored the expression of IL-33 and sST2 in mice with STZ-induced DN and found that IL-33 was significantly increased in both DN model mice and DN patients. Current studies have primarily focused on podocytes and the pathogenesis of proteinuria and glomerulosclerosis. However, endothelial cells have largely been overlooked in the context of glomerular injury. The localization of IL-33 in the kidney was determined to be mainly concentrated in the glomeruli, blood vessels, and peritubular capillaries [17]. In this study, co-staining with the endothelial cell marker CD31 demonstrated that IL-33 was mainly localized on GECs. Our renal functional analysis and HE staining revealed that the treatment with IL-33 attenuated renal injury in mice with STZ-induced DN, and these results are consistent with the evidence described above [26]. IL-33 has been reported to significantly reduce FBG levels in HFD mice. In our study, although there was no significant difference, the FBG level in DN mice tended to decrease after treatment with IL-33 [27]. This may suggest an unexplored potential role for IL-33 in hypoglycemia.

ER stress is a crucial inducer of cell apoptosis under physiological and pathological conditions [17, 28] and acts as an indispensable factor in the pathogenesis of DN [30]. Therefore, reducing ER stress may hinder the progression of DN. Activation of three sensors, PERK, ATF6, and IRE1, by ER stress is negatively regulated by GPR78/BiP [31]. Under balanced homeostasis, ER stress sensors form an inactive complex with GRP78. Under ER stress conditions, increased binding of the ER companion GPR78 to unfolded proteins results in the dissociation of GPR78 from the ER stress sensors PERK, IRE1, and ATF6, leading to their activation. IL-33 has been proven to exert neuroprotective effects against RNS-induced brain injury by inhibiting apoptosis, ER stress, and inflammatory pathways [32]. Our results demonstrated that IL-33 relieves renal ER stress by downregulating the expression of PERK and IRE1 in DN model mice but does not affect the expression of ATF6. PERK and IRE1 are fundamental branches of the UPR, are partially similar, and are both type 1 transmembrane proteins [33]. Unlike PERK and IRE1, ATF6 is a type 2 transmembrane protein with a carboxy-terminal stress-sensing luminal domain [34]. We speculate that ATF6 is not regulated by IL-33 may be due to the fact that ATF6 has different sensors than PERK and IRE1 when responding to IL-33. Moreover, IL-33 stimulation effectively suppressed HG-induced activation of GPR78, CHOP, PERK, and IRE1 in MGECs. A study demonstrated that apoptosis of glomerular endothelial cells can be mediated by the ER stress pathways PERK and IRE1 [35]. In this study, flow cytometric results showed that MGEC apoptosis increased exponentially under HG conditions but was reduced by IL-33 stimulation. In addition, sST2 stimulation alone did not alter the levels of those proteins in the HG environment, but co-stimulation with IL-33 reversed the regulatory effects of IL-33. Furthermore, sST2 pretreatment obviously reversed the role of IL-33 in inhibiting ER stress and apoptosis, suggesting that IL-33 might suppress ER stress or apoptosis, at least in part, via the sST2 signaling pathway following DN.

In conclusion, the current data demonstrate that IL-33 ameliorates DN-induced renal injury by inhibiting ER stress and apoptosis. In parallel, sST2 treatment significantly reversed these effects of IL-33. We suggest that IL-33 may be an effective therapeutic target for DN.

### Electronic supplementary material

Below is the link to the electronic supplementary material.

**Supplementary Material 1:** Raw experimental data

## Data Availability

All data generated or analyzed during this study are included in this published article.

## Abbreviations

DN: Diabetic nephropathy
DM: Diabetes mellitus
ER stress: Endoplasmic reticulum stress
STZ: Streptozotocin
ESRD: End-stage renal disease
IL-33: Interleukin − 33
AKI: Acute kidney injury
CKD: Chronic kidney disease
GPR78: Glucose-regulated protein 78
HG: High glucose
FBG: Fast blood glucose
UCE: Urinary creatinine excretion
UPR: Urine protein
UPC: Urine protein creatinine ratio
NG: Normal glucose
