# Supplementary material for: IL-33/ST2L signaling alleviates diabetic nephropathy by regulating endoplasmic reticulum stress and apoptosis
Source: BMC Nephrol. 2023 Dec 5;24:361. doi: 10.1186/s12882-023-03415-8 (PMC10698915; doi:10.1186/s12882-023-03415-8)

S1. Raw data

Densitometry analysis of Western blots (Figure 4A) showing total caspase-12, CHOP, GAPDH, GPR78, IREK, PERK, p-IREK, p-PERK expression in mouse glomerular endothelial cell lines. GAPDH was used as a loading control. All data determined in triplicate mean ± standard deviation. * p<0,05; **p<0.01 ; ***p<0.001 (Student’s t-test).

1.
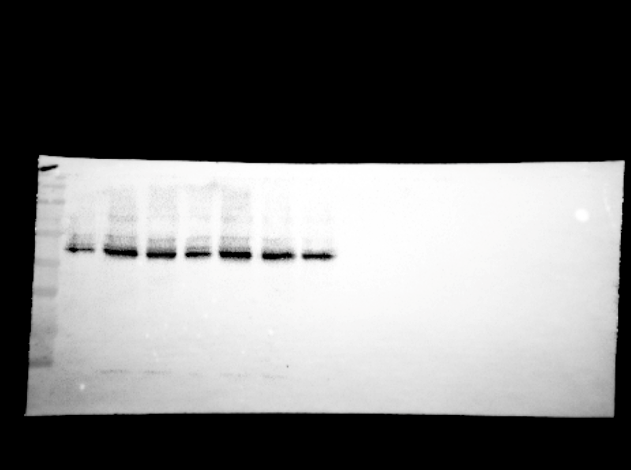

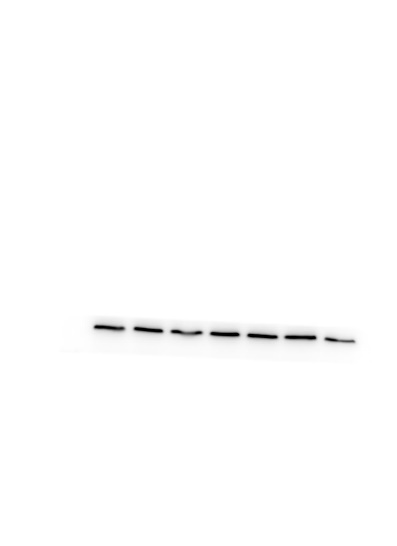

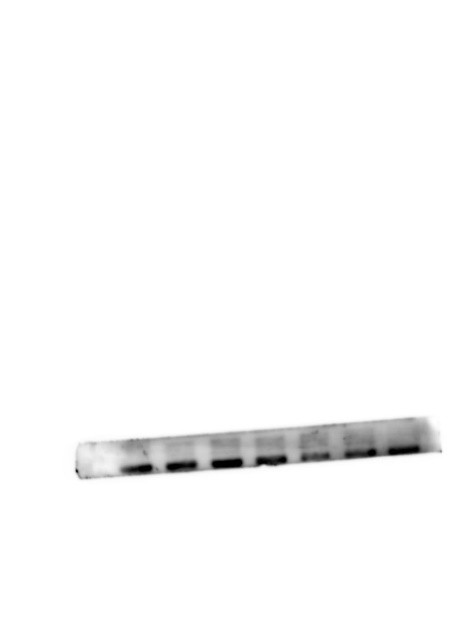
caspase-12
2.
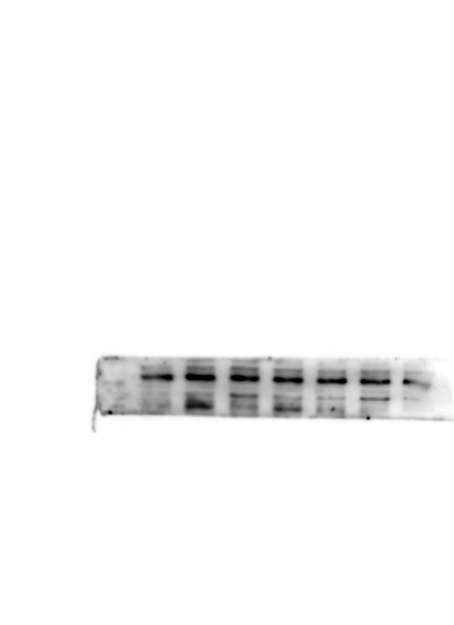
CHOP


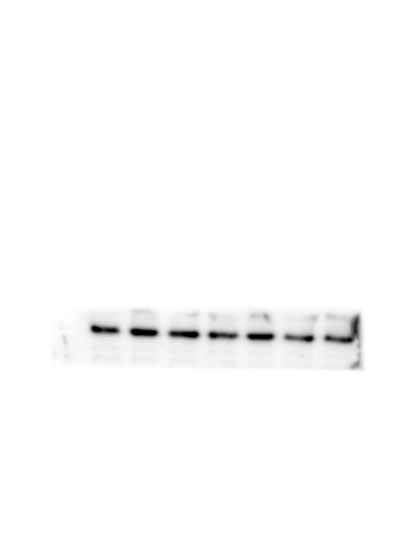


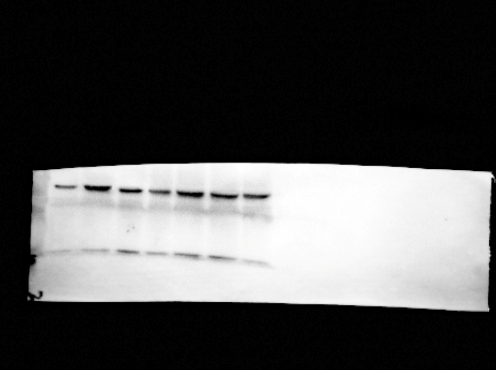


1.
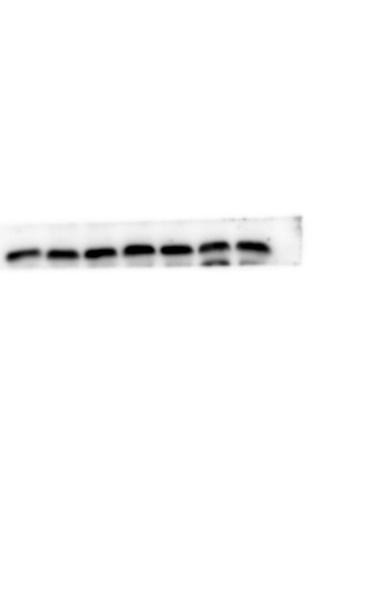
GAPDH


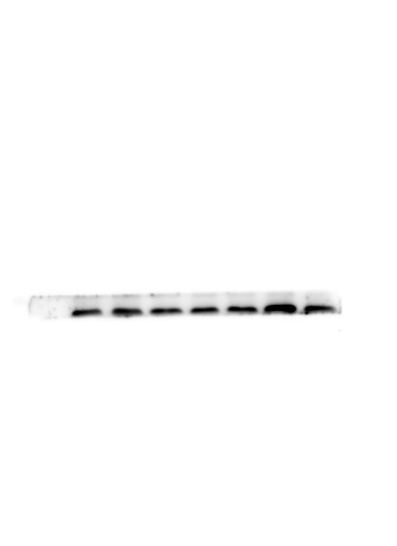


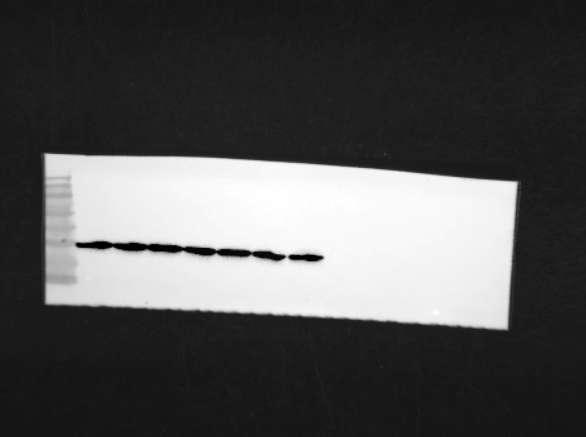


1. GPR78


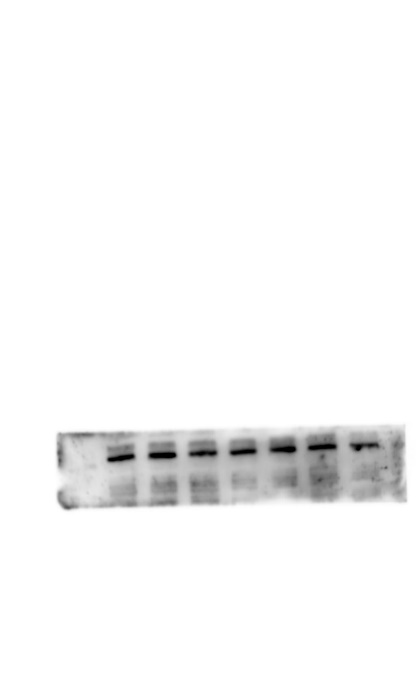


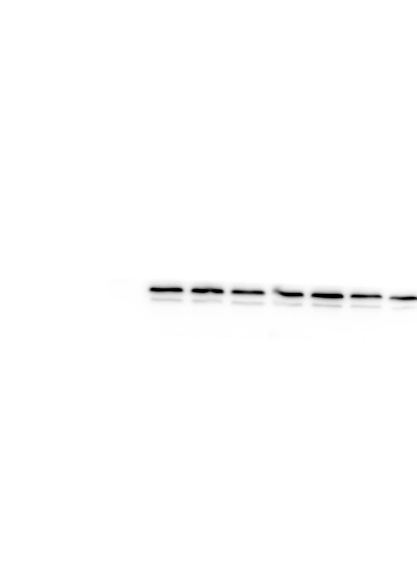


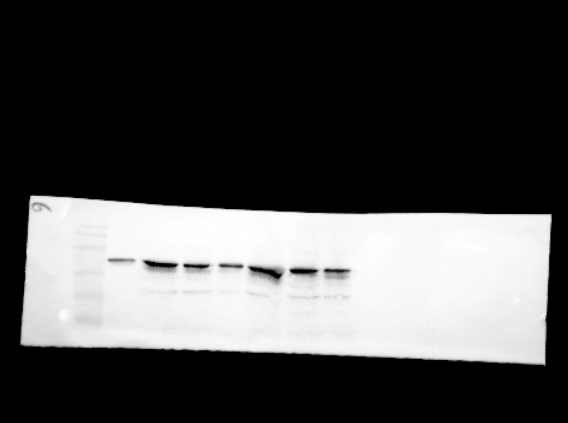


1. IREK


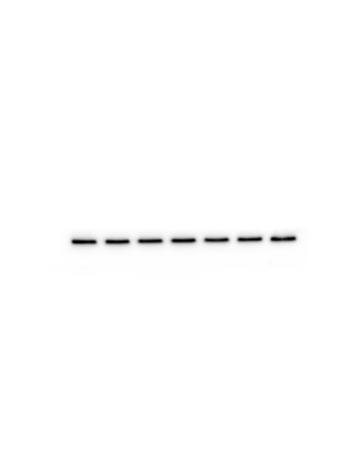

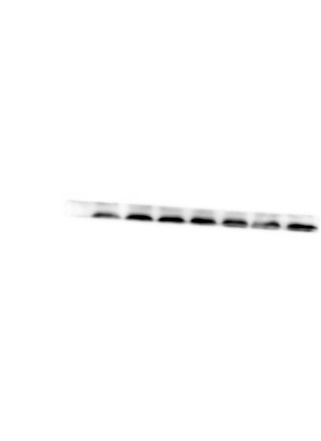


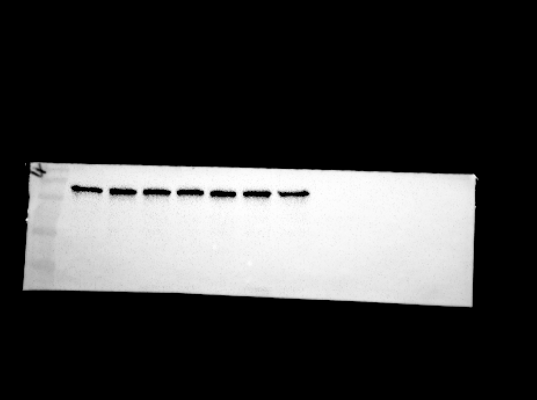


1. PERK
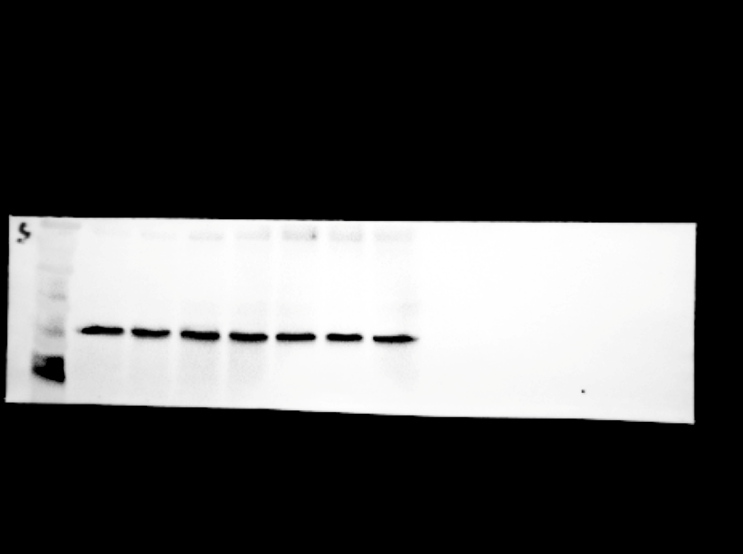


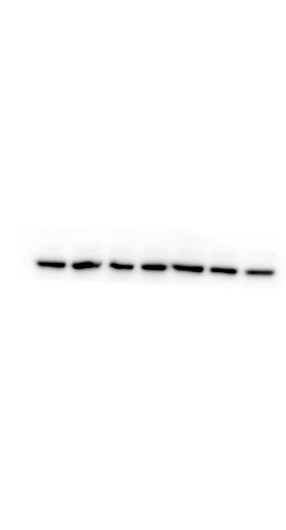


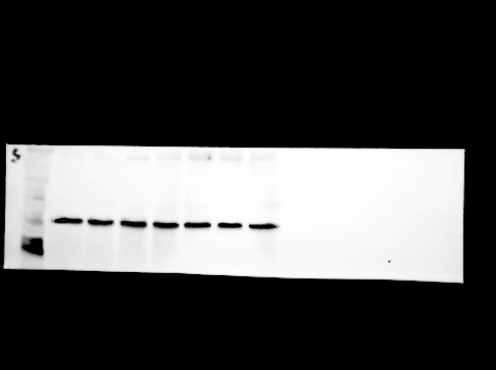

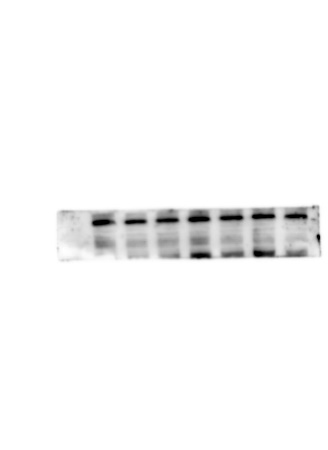


G. p-IREK


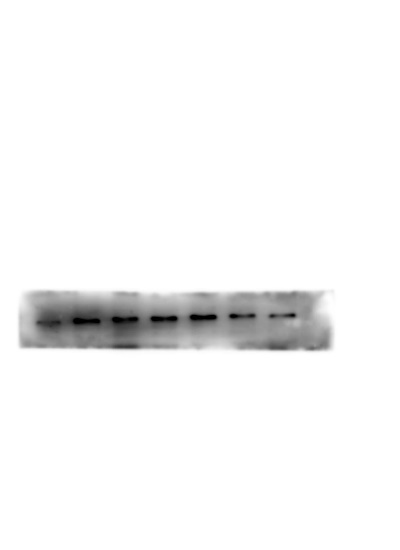


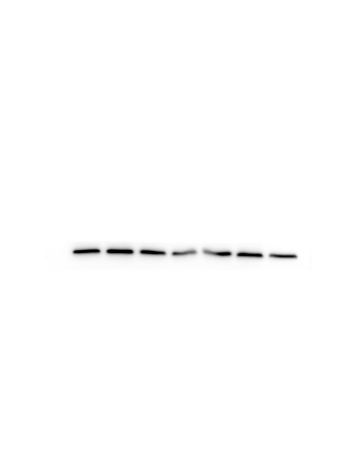

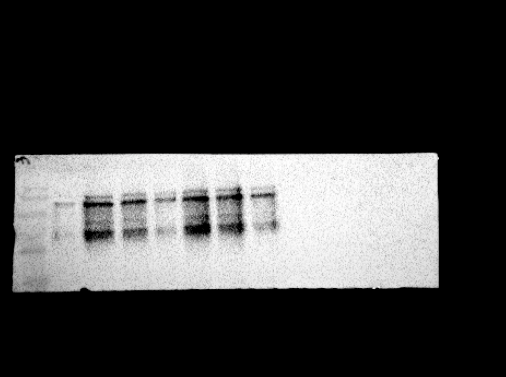


1. p-PERK


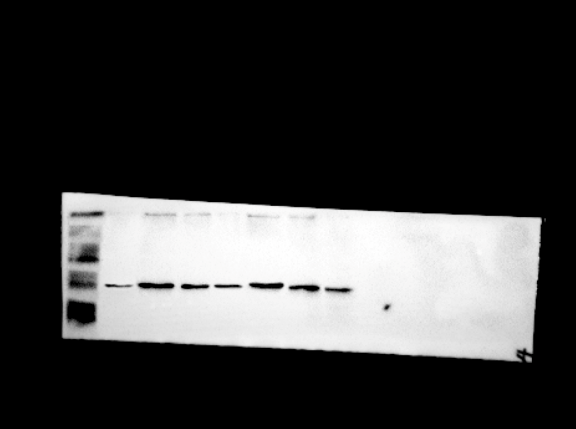


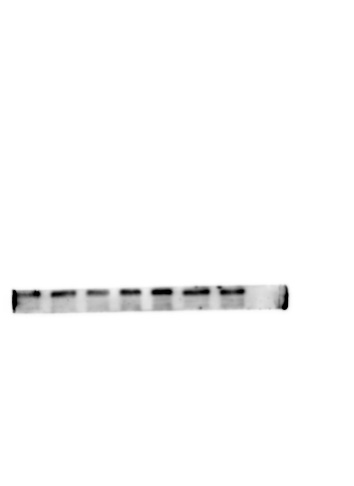

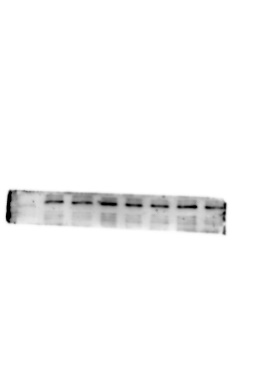

Supplement: Supplementary file 1 — Supplementary Material 1: Raw experimental data [file 12882_2023_3415_MOESM1_ESM.docx]
